# Aleocharine rove beetles (Coleoptera, Staphylinidae) associated with *Leptogenys* Roger, 1861 (Hymenoptera, Formicidae) I. Review of three genera associated with *L. distinguenda* (Emery, 1887) and *L. mutabilis* (Smith, 1861)

**DOI:** 10.3897/zookeys.59.510

**Published:** 2010-10-01

**Authors:** Munetoshi Maruyama, Christoph von Beeren, Rosli Hashim

**Affiliations:** 1 The Kyushu University Museum, Fukuoka, 812-8581 Japan; 2 Department Biologie II, Ludwig-Maximilians-Universität München, Großhaderner Straße 2, 82152 Planegg-Martinsried, Germany; 3 Institute of Biological Science, Faculty of Science, University of Malaya, 50603 Kuala Lumpur, Malaysia

**Keywords:** Myrmecophily, Maschwitzia, Togpelenys, Witteia gen. n., Wroughtonilla genus group, new species, new combination, Malaysia, new host record

## Abstract

Three myrmecophilous genera of Aleocharinae (Staphylinidae) associated with Leptogenys distinguenda (Emery, 1887) and Leptogenys mutabilis (Smith, 1861) are reviewed with descriptions of new taxa: Maschwitzia Kistner, 1989, Togpelenys Kistner, 1989 and Witteia Maruyama & von Beeren, **gen. n.** (type species: Witteia dentilabrumMaruyama & von Beeren, **sp. n.**). The following new combinations are proposed: Zyras (s. lat.) aenictophila (Kistner, 1997),**comb. n.** (*ex* Maschwitzia), Zyras (s. lat.) dichthadiaphila (Kistner in Kistner et al., 2003), **comb. n.** (*ex* Maschwitzia), Maschwitzia derougemonti (Pace, 1984), **comb. n.** (*ex* Wroughtonilla Wasmann, 1899), Maschwitzia watanabei (Maruyama, 2004), **comb. n.** (*ex* Wroughtonilla), Maschwitzia dilatata (Pace, 2005), **comb. n.** (*ex* Wroughtonilla), Witteia borneensis (Pace, 1986), **comb. n.** (*ex* Wroughtonilla). These genera belong to the Wroughtonilla genus group of the tribe Lomechusini.

## Introduction

The ant genus Leptogenys Roger, 1861 belongs to the subfamily Ponerinae. Some of its members show army ant-like behavior (Maschwitz et al. 1989, Kronauer 2009). Many Leptogenys species harbor various groups of myrmecophilous insects comparable to the myrmecophile richness of the classic army ants of the subfamilies Dorylinae, Aenictinae and Ecitoninae (Witte et al. 2008). Rove beetles associated with Leptogenys ants have been studied by several authors based on the material collected by ant researchers (Wasmann 1899; Kistner 1975, 1989; Kistner et al. 2008; Hlaváč and Janda 2009). The rove beetles associated with Leptogenys ants show strict host-species specificity, i.e. one rove beetle species is associated with only one host ant species (Maruyama, unpublished data; von Beeren and Witte, personal observations). In this article we present the first known exception to this rule, with Maschwitzia ulrichi Kistner, 1989 occurring in two closely related Leptogenys host species. The already described species, Maschwitzia ulrichi and Togpelenys gigantea Kistner 1989 were recorded from colonies of Leptogenys distinguenda (Emery, 1887) at Ulu Gombak in Peninsular Malaysia (Kistner 1989). Although the former species was recorded from a Leptogenys borneensis colony (Kistner et al. 2008), this is most probably based on a misidentification (Maruyama et al. 2010). Recently, the junior author (CvB) collected a series of rove beetles from Leptogenys distinguenda colonies and from one Leptogenys mutabilis (Smith, 1861) colony in Peninsular Malaysia. The material included an unknown species with an autapomorphy, which did not allow it to be assigned to any known genus.

In the present article, we revise some of the rove beetle genera associated with Leptogenys ants. This first part of the series reviews the genera which are associated with Leptogenys distinguenda and Leptogenys mutabilis, including descriptions of some new taxa and some new combinations.

## Materials and methods

The rove beetles were collected in spring and autumn 2008 and 2009 in a well regenerated dipterocarp rainforest in the Field Studies Centre of the University of Malaya in Ulu Gombak, Malaysia (03°19.4796N; 101°45.1630E, altitude 230 m) and near the Institute of Biodiversity in Bukit Rengit, Malaysia (03°35.779N; 102°10.814E, altitude 72 m). Nests of the nocturnal host ants were located during the night by back-tracking Leptogenys raiding trails. Since all rove beetles follow the host ant migrations, we detected them on these occasions and collected them with aspirators (for further information see Witte et al. 2008). The specimens were put in 1.5 ml plastic tube containing 80 % ethanol for morphological studies.

The methods of dissecting and line drawings followed Maruyama (2006). Dissected genitalia and mouthparts were mounted in Euparal on a small glass plate (10 × 5 mm), and subsequently glued onto a paper card (6 × 5 mm) and pinned under the respective specimen (Maruyama 2004). Photographs were taken with a Canon EOS Kiss X1 with a macro lens MP-E 65, and then combined by the automontage software CombineZM. Specimens are deposited in the senior author’s collection in the Kyushu University Museum (KUM) and in the Bavarian State Collection of Zoology (Munich, Germany). Measurements are given in millimeters and are abbreviated as follows: antennal length (AL); body length (BL); fore body length, from front margin of head to apices of elytra (FBL); hind tibial length (HTL); head length (HL); head width (HW); pronotal length (PL); pronotal width (PW).

**Figures 1–4.**
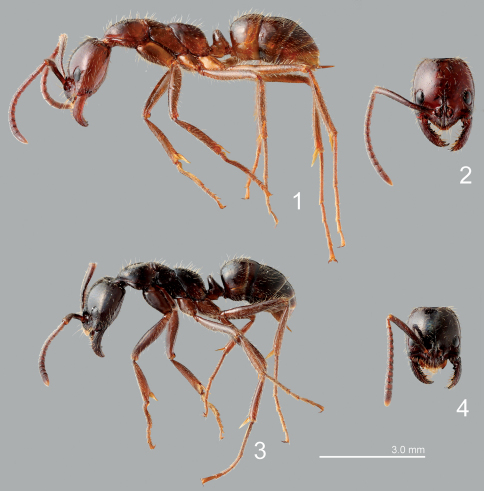
Host ants. **1**  Leptogenys distinguenda, lateral view **2** ditto, head **3** Leptogenys mutabilis, lateral view **4**  ditto, head.

Leptogenys distinguenda is sometimes treated as a subspecies of Leptogenys processionalis Jerdon, 1851 known from India (Emery 1911; Bolton 1995). However, the taxonomy of Leptogenys species in Asia has been poorly studied, and identifications of the known species remain confusing (Ito pers. comm.). We tentatively follow the current papers citing Leptogenys distinguenda as a distinct species (e.g., Witte and Maschwitz 2000), until a revisional study of all Asian Leptogenys species is completed. Both of the antspecies in the present paper are illustrated (Figs 1–4) to specify our identifications of the species.

## Taxonomy

### Maschwitzia

Kistner, 1989

Fig. 5

Maschwitzia Kistner 1989: 301 (original description).

#### Type species.

Maschwitzia ulrichi Kistner, 1989.

#### Diagnosis.

 This genus is closely allied to Witteia in general appearance, especially pronotal shape, but may easily be distinguished from it by having a generalized labrum, not strongly sclerotized and without projections; the simple mandibles, their inner edges not emarginate at middle; the straight lateral projections of the labial apodeme; the much smaller eyes; the shorter antennae; and the shorter legs.

**Figures 5–8.**
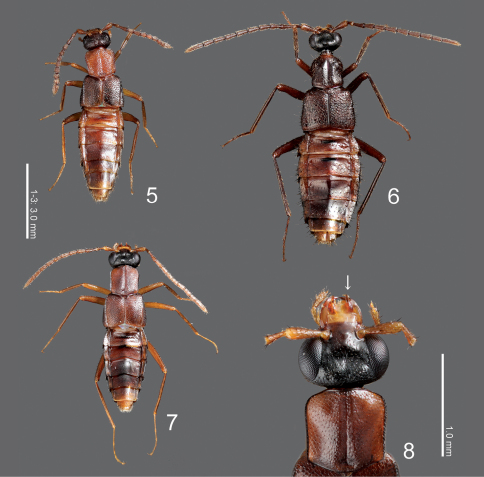
Type species of the genera Maschwitzia, Togpelenys and Witteia **5**  Maschwitzia ulrichi, dorsal habitus **6**  Togpelenys gigantea, dorsal habitus **7**  Witteia dentilabrum gen. et sp. n., dorsal habitus **8**  ditto, head and pronotum, dorsal view.

#### Comments.

Kistner et al. (2008) transferred Trachydonia aenictophila Kistner, 1997 and Trachydonia dichthadiaphila Kistner, 2003 to Maschwitzia. However, they are apparently not members of Maschwitzia, nor even closely related in view of the absence of the autapomorphies of Wroughtonilla Wasmann, 1899 and its allied genera (see Discussion). Though the genus Zyras Stephens, 1835 is heterogeneous, apparently non-monophyletic and not well defined yet, they can be placed in Zyras (s.lat.) by sharing the general diagnostic features of the genus (e.g., Fenyes 1920) and excluded from Maschwitzia, as follows:

Zyras (s. lat.) aenictophila (Kistner in Kistner et al. 1997), **comb. n.**

Zyras (s. lat.) dichthadiaphila (Kistner in Kistner et al. 2003), **comb. n.**

Trachydonia Bernhauer 1928 has been placed as a subgenus of Zyras, but Kistner et al. (2003) raised it to generic status. At least Zyras aenictophila and Zyras dichthadiaphila are not members of Trachydonia. They are also not members of the subgenus Zyras. The genus Zyras should be subdivided into several genera based on a phylogenetic analysis, and then adequate systematic affiliation of these two species may be found.

The following four species are known in Maschwitzia.

#### Maschwitzia ulrichi

Kistner, 1989

Fig. 5

Maschwitzia ulrichi Kistner 1989: 307 (original description).
Trachydonia leptogenophila  Kistner in Kistner et al. 2003: 386 (original description); Maruyama 2004: 96 (synonymized with Maschwitzia ulrichi).

##### Type locality.

 Ulu Gombak, Selangor, Malaysia.

##### Additional records.

 Ulu Gombak (University Malaya Field Studies Centre, 03°19.479N; 101°45.170E, 220–250 m alt.), Selangor, Malaysia, VIII 2008, C. von Beeren from the colony of Leptogenys distinguenda (10); same data, but III 2009, C. von Beeren and V. Witte (12); same data, but VIII 2009, C. von Beeren (10); same data, but IX 2009, Y. Nakase (6); same data, but III 2010, C. von Beeren from the colony of Leptogenys mutabilis (2); Bukit Rengit, Pahang, Malaysia (03°35.779N; 102°10.814E, altitude 72 m): C. von Beeren and V. Witte (8).

##### Distribution.

Peninsular Malaysia.

##### Symbiotic hosts.

Leptogenys distinguenda and Leptogenys mutabilis.

##### Diagnosis.

This species is closely similar to Maschwitzia watanabei in general appearance, but is distinguished from it by the pronotum being narrower around the posterior margin and the aedeagus being different in shape, especially with the apical part of the median lobe being strongly widened apically and not excavated paramerally.

##### Comments.

Two specimens were collected for the first time from the end of a migration column of Leptogenys mutabilis (new host record).

#### Maschwitzia derougemonti

(Pace, 1984) comb. n.

Wroughtonilla derougemonti Pace 1984: 460 (original description).

##### Type locality.

 Kalaw, Myanmar

##### Distribution.

Myanmar.

##### Symbiotic host.

Unknown.

##### Diagnosis.

The aedeagal shape is clearly different from the other congeners in particular the parameral crest is larger and the apical lobe longer.

##### Comments.

Only the holotype and one paratype are known. The original description by Pace (1984) agrees well with the characteristics of the other congeners. The symbiotic host is probably, Leptogenys distinguenda or its related species. However, Asian species of Leptogenys are in need of revision, and distributions of most known species, including Leptogenys distinguenda, are still uncertain.

#### Maschwitzia watanabei

(Maruyama, 2004) comb. n.

Wroughtonilla watanabei Maruyama 2004: 92 (original description).

##### Type locality.

Bolikhamsai (Borikhamxay), Laos.

##### Distribution.

Laos.

##### Symbiotic host.

Unknown.

##### Diagnosis.

This species is closely similar to Maschwitzia ulrichi in general appearance, but is distinguished from it by the pronotum being wider around the posterior margin and by the different shape of the aedeagus, especially the apical part of the median lobe being less widened apically and largely excavated paramerally.

##### Comments.

Only the holotype is known. The symbiotic host is probably Leptogenys distinguenda or a related species, although Leptogenys distinguenda is not recorded from Laos at present.

#### Maschwitzia dilatata

(Pace, 2005) comb. n.

Wroughtonilla dilatata Pace 2005: 147 (original description).

##### Type locality.

Umran, East Khasi Hills, Meghalaya, India.

##### Distribution.

Meghalaya, India.

##### Symbiotic host.

Unknown.

##### Diagnosis.

This species is closely similar to Maschwitzia ulrichi in general appearance, but is distinguished from it by the pronotum being wider around the posterior margin and by the different shape of aedeagus, especially the apical part of the median lobe being less widened apically and largely excavated paramerally.

##### Comments.

Only the holotype has been known. The original description by Pace (2005) agrees well with the characteristics of the other congeners and he noted that this species is allied to Maschwitzia derougemonti. The symbiotic host is probably Leptogenys distinguenda or a related species.

### Togpelenys

Kistner, 1989

Fig. 6

Togpelenys Kistner 1989: 308 (original description).

#### Type species.

Togpelenys gigantea Kistner, 1989.

#### Diagnosis.

This genus is clearly distinguished from the other genera of Wroughtonilla group by the combination of the following character states: eyes extremely large; pronotum without superior marginal line of the pronotal hypomeron; pronotal disc quite convex, with a shallow and large longitudinal depression; pronotum and elytra covered with long, suberect macrosetae; and abdomen large, expanded, much wider than elytra.

#### Distribution.

Peninsular Malaysia.

#### Comments.

 Only the type species Togpelenys gigantea Kistner, 1989 has been known in the genus. Probably further species will be found from the regions around Peninsular Malaysia, e.g., Sumatra, Borneo and Java.

#### Togpelenys gigantea

Kistner, 1989

Fig. 6

Togpelenys gigantea Kistner 1989: 312 (original description).

##### Type locality.

 Ulu Gombak, Selangor, Malaysia.

##### Additional record.

Bukit Rengit (03°35.779N; 102°10.814E; 72 m alt.), Pahang, Malaysia, III 2009, C. von Beeren and V. Witte (2 males).

##### Distribution.

Peninsular Malaysia.

##### Symbiotic host.

Leptogenys distinguenda.

##### Diagnosis.

This species is easily distinguished from the other species of Wroughtonilla as well as its allied genera by the generic diagnosis.

##### Comments.

 Rare species, newly recorded from Pahang. In the type locality, Ulu Gombak, Selangor, no additional specimen has been collected despite more than 40 colonies having been examined in the last few years (von Beeren and Witte, personal observations; the type series were collected in 1982). We are not sure whether this is due to environmental changes in Ulu Gombak or simply due to rarity of this species.

### Witteia

Maruyama & von Beeren gen. n.

urn:lsid:zoobank.org:act:B3D89EA2-4867-40F5-9817-E6E108D0BA2B

Figs 7 18 

#### Type species.

 Witteia dentilabrum sp. n.

#### Etymology.

 Dedicated to Dr. Volker Witte for his contribution to the biology of Leptogenys ants and their symbionts. Gender, feminine.

#### Diagnosis.

This genus is similar to Maschwitzia Kistner, 1989 in body shape and punctation of body surface, but may easily be distinguished from it by the labrum being strongly sclerotized and with a pair of spines; the inner margins of the mandibles emarginate at middle; the lateral projections of the labial apodeme curved apically; the extremely large eyes; the longer antennae; and the longer legs.

#### Description.

##### Body

 (Fig. 7) elongate, flattened; surface of fore body (Fig. 8) weakly rugose, reticulated, somewhat matte.

##### Head

**Figures 9–13.**
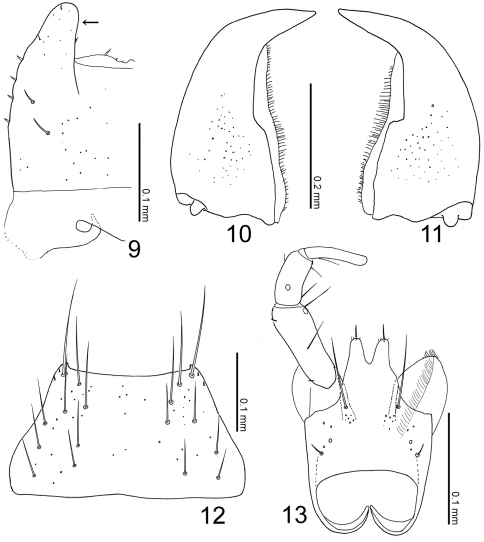
Mouthparts of Witteia dentilabrum gen. et sp. n. **9** labrum, left side, dorsal view **10** left mandible, dorsal view **11** right mandible, dorsal view **12** mentum, ventral view **13** labium, ventral view.

 (Figs 7–8) transverse, with eyes extremely large, somewhat shorter than head, with a round depression above; clypeus truncate apically. Labrum (Figs 8–9) strongly sclerotized, with a pair of projections laterally (Figs 9: arrow). Mandibles asymmetrical, strongly curved, each apex acutely pointed; inner margin of left mandible (Fig. 10) shallowly emarginate; that of right (Fig. 11) rather largely emarginated. Mentum (Fig. 12) trapezoidal, with several thick setae, with sparse pseudopores. Labium (Fig. 13) broad; prementum with a setal pore and 2 real pores near base, with several pseudopores around inner ridges; apodeme without median projection, with lateral projection curved apically; ligula long, each lobe with a large setulum and three small setula; labial palpus with segment I long and apically dilated; segment II half as long as I; segment III thin, parallel-sided, slightly shorter than II.

##### Pronotum

 (Figs 7–8) with disc well margined, slightly convex, with a narrow longitudinal groove medially and a pair of depressions postero-laterally. Mesocoxal cavity well margined; process of metaventrite narrow, pointed at apex.

##### Elytra

 (Fig. 7) apically widened, laterally with a pair of rather high carinae, that are slightly curved inwards.

##### Legs

 (Fig. 7) very long, thin; femora slightly narrowed apically near apex; tibiae somewhat widened around middle to basal 1/3, their bases constricted; tibiae very thin, filiform.

##### Abdomen

 (Fig. 7) fusiform, flattened, widest around apex of segment III; surface smooth, sparsely punctured, shining. Median lobe of aedeagus (Figs 16–17) with long and narrow apical part, with a small projection near base of apical part (Fig. 16: arrow). Paramere with apical lobe slightly widened apically.

**Figures 14–18.**
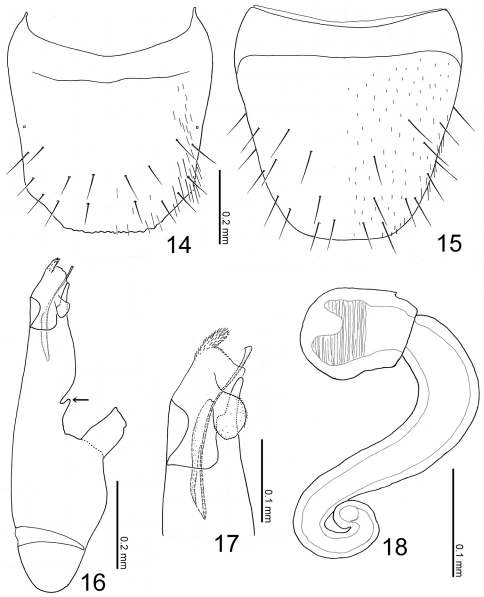
Terminalia of Witteia dentilabrum gen. et sp. n. **14** Male tergite VIII, dorsal view **15** male sternite VIII, ventral view **16** median lobe of aedeagus, lateral view **17** ditto, apical part **18** spermatheca.

#### Witteia dentilabrum

Maruyama & von Beeren sp. n.

urn:lsid:zoobank.org:act:EF84731E-0E8D-486B-859B-DBD89510FC8A

Figs 7 18 

##### Etymology.

Referring to the lateral projections on the labrum which is a unique character state in Witteia.

##### Type series.

 Holotype, male, Ulu Gombak (University Malaya Field Studies Centre, 03°19.479N; 101°45.170E, 220–250 m alt.), Selangor, Malaysia, III 2009, C. von Beeren and V. Witte, from a colony of Leptogenys distinguenda (KUM). Paratypes: same data as holotype (2 males, 1 female); same data, but VIII 2008, C. von Beeren (10); same data, but VIII 2008, C. von Beeren (8); same data, but IX 2009, Y. Nakase (2).

##### Type locality.

Ulu Gombak, Selangor, Malaysia.

##### Distribution.

Peninsular Malaysia.

##### Symbiotic host.

Leptogenys distinguenda.

##### Diagnosis.

This species is closely similar in general appearance to Witteia borneensis (Pace, 1986), comb. n., from Sabah, Borneo, but is distinguished from it by the larger body and the smaller apical part of the spermatheca.

##### Description.

###### Body

 (Fig. 7) color reddish brown, but head completely black, mouthparts, legs, apex of abdomen lighter, medial areas of abdominal segments V and VI infuscate. Head (Figs 7–8) sparsely covered with setae; surface finely reticulated. Antennae (Figs 7–8) long, filiform; all segments longer than wide; segments III-X almost twice as long as wide; segment XI elongate. Pronotum (Figs 7–8) subquadrate, subparallel-sided, slightly wider than long (width/length = 1.11–1.18); surface moderately covered with minute setae, with some minute macrosetae laterally. Abdomen with anterior margins of sternites IV-VI produced medially; tergite VIII (Fig. 14) crenulate apically, with 8 macrosetae; sternite VIII (Fig. 15) rounded apically.

###### Male:

 sternite VIII with 11–12 macrosetae. Median lobe of aedeagus (Figs 16–17) with large parameral crest; apical part roundly convex paramerally; apical lobe slightly trilobed; copulatory piece with a short flagellum.

###### Female:

 sternite VIII with 8–9 macrosetae. Spermatheca (Fig. 18) with basal part dilated apically, coiled near base, curved near apex; apical part short.

###### Measurements:

 BL, ≈ 4.2–5.1; FBL, ≈ 1.8–2.0; HL, 0.660–0.738; HW, 0.887–0.988; AL, ≈ 2.8–3.1; PL, 0.806–0.950; PW, 0.725–0.800; HTL, 1.238–1.438.

##### Comments.

 Commonly found in Leptogenys distinguenda colonies together with Maschwitzia ulrichi, but less frequent than the latter species.

#### Witteia borneensis

(Pace, 1986) comb. n.

Wroughtonilla borneensis Pace 1986: 204.

##### Type locality.

Pangi, Sabah, Malaysia.

##### Diagnosis.

This species is closely similar in general appearance to Witteia dentilabrum sp. n. from Selangor, Malaysia, but is distinguished from it by the smaller body (3.0 mm) and the larger apical part of the spermatheca.

##### Distribution.

 Sabah, Borneo; Burma.

##### Comments.

 This species was described based on a single specimen from Sabah, Borneo. In the original description Pace (1986) illustrated the habitus and spermatheca. The habitus illustration shows the labrum with a pair of projections that is an autapomorphy of Witteia. This species is probably associated with Leptogenys distinguenda.

## Discussion

The genera Maschwitzia, Togpelenys and Witteia are closely allied to Wroughtonilla (one species from India, Sri Lanka, Malaysia) and they belong to the Wroughtonilla genus-group (here proposed) of the subtribe Myrmedonina of the tribe Lomechusini, together with the genera Aenictonia Wasmann, 1900 (10 species from tropical Africa, one species from Thailand), Anommatochara Wasmann, 1915 (one species from tropical Africa), Leptogenoxenus, 1975 (one species from Philippines) and Neowroughtonilla, 1989 (one species from Malaysia). All members of this genus-group are associated with Leptogenys ants as far as known, except for species of Aenictonia and Anommatochara  which are associated with Dorylus Fabricius, 1793 and/or Aenictus Shuckard, 1840 ants. The genera of this group share the following apomorphic character states: head with “neck”, a constricted postoccipital suture; pronotum with a longitudinal median groove; elytra with a pair of carinae laterally; and apical lobe of aedeagal median lobe elongate.

When Hlaváè and Janda (2009) described the genus Leptogenopapus (species from Papua New Guinea, associated with Leptogenys breviceps Viehmeyer, 1914), they stated that it is closely related to Leptogenoxenus. However, Leptogenopapus does not share the character states mentioned above. Because the type species Leptogenopapus mirabilis is in its general appearance extremely modified for myrmecomorphy, it is possible that the apomorphic character states in the Wroughtonilla group cited above have been secondarily lost or modified in this species. However, the aedeagal shape, which is normally not modified along with modification of external morphology to the myrmecophilous habitat, of Leptogenopapus mirabilis is very different from those of the Wroughtonilla group. Leptogenopapus does not belong to the Wroughtonilla genus-group.

Witteia is established as a new genus due to a strong autapomorphy, the presence of the projections on the labrum, by which it is clearly distinguished from Maschwitzia which is similar overall in general appearance. This character state is unique in the Lomechusini, possibly not present in any other aleocharine genera. Several other character states further distinguish Witteia from Maschwitzia (see Diagnosis of both the genera), though their polarities remain uncertain. We were reluctant to establish Witteia based on this single autapomorphy. However, considering generic concepts of the Wroughtonilla genus-group, which seem relatively narrow, the present establishment of the new genus seems reasonable at present. Phylogeny-based, ideally molecular phylogeny-based systematic revisions will be needed in the future.

Leptogenys distinguenda has been known as the only host of Maschwitzia ulrichi. However, it was collected also from colonies of Leptogenys mutabilis (new record above). This is the only confirmed example of Leptogenys-associated aleocharine species that parasitizes more than one species of Leptogenys ants. Leptogenys distinguenda and Leptogenys mutabilis are morphologically very similar which suggests their close phylogenetic relationships, and this probably allows Maschwitzia ulrichi to parasitize both species. In other myrmecophilous aleocharine species of Lomechusini, some of the Asian species of the genus Pella Stephens, 1835 have been known to host more than one ant species of Lasius spp. that are also very closely related, i.e., they belong to the same subgenus (Maruyama 2006).

## Supplementary Material

XML Treatment for Maschwitzia

XML Treatment for Togpelenys

XML Treatment for Witteia
